# Biological attributes of the kissing bug *Triatoma rubrofasciata* from Vietnam

**DOI:** 10.1186/s13071-019-3844-6

**Published:** 2019-12-16

**Authors:** Ho Viet Hieu, Le Thanh Do, Sebastián Pita, Hoang Ha, Pham Thi Khoa, Pham Anh Tuan, Ta Phuong Mai, Ngo Giang Lien, Francisco Panzera

**Affiliations:** 1 Department of Medical Microbiology and Parasitology, Faculty of Medicine, Duy Tan University, Da Nang, 550000 Vietnam; 2 Institute for Global Health Innovations, Duy Tan University, Da Nang, 550000 Vietnam; 3 Sección Genética Evolutiva, Facultad de Ciencias, Universidad de la República, 11400 Montevideo, Uruguay; 4 Department of Cell Biology, University of Science, Vietnam National University, Hanoi, 100000 Vietnam

**Keywords:** Asia, Blood-sucking bug, *Triatoma rubrofasciata*, *Trypanosoma conorhini*, *Trypanosoma lewisi*, Vietnam

## Abstract

**Background:**

*Triatoma rubrofasciata* is the only kissing bug species distributed globally. In the Americas, this species transmits the parasite *Trypanosoma cruzi*, responsible for Chagas disease. The presence of *T. rubrofasciata* in several Asian countries has greatly increased recently. In Vietnam, it is found in large numbers, closely associated with human environments. Although *T. rubrofasciata* from Asia is not infected with *Tryp. cruzi*, it carries other parasites such as *Trypanosoma lewisi* and *Trypanosoma conorhini.* Reports of bites by *T. rubrofasciata* have increased significantly in several places of Vietnam, becoming a public health problem as it produces severe anaphylactic reactions.

**Methods:**

Specimens of *T. rubrofasciata* were collected from seven provinces in central Vietnam. We analyzed different biological attributes (life-cycle, starvation resistance, feeding and reproductive capacities) and genetic characteristics (chromosomes and DNA sequences) of *T. rubrofasciata* from Vietnam and compared them with Brazilian specimens. Natural infection with *Tryp. conorhini* and *Tryp. lewisi* were analyzed in a sample of 100 collected insects.

**Results:**

Species identification of *T. rubrofasciata* from central Vietnam was corroborated by genetic markers. Cytogenetic analyses showed that *T. rubrofasciata* from central Vietnam share the same chromosomal characteristics with individuals from Brazil and Hanoi. DNA sequence analyses of a mitochondrial *cytochrome b* gene fragment showed little variation between Old and New World specimens. Our study sample, compared with Brazilian individuals, showed a higher survival capacity revealed by a higher hatching rate (98% compared with 80.5%), a larger amount of blood taken in single meal and long-term starvation resistance. Furthermore, this species had a high natural rate of infection with *Tryp. conorhini* (46%) and *Tryp. lewisi* (27%).

**Conclusions:**

For *T. rubrofasciata* of Vietnam, a high rate of fecundity throughout the year, a high capacity for starvation, and its occurrence in synanthropic environments of urban areas with a high availability of food sources are risk factors to be taken into account by vector control campaigns. The several allergic reactions caused by their bites and their high infection with *Tryp. lewisi* highlight the need to implement specific control programmes for *T. rubrofasciata* in Vietnam.
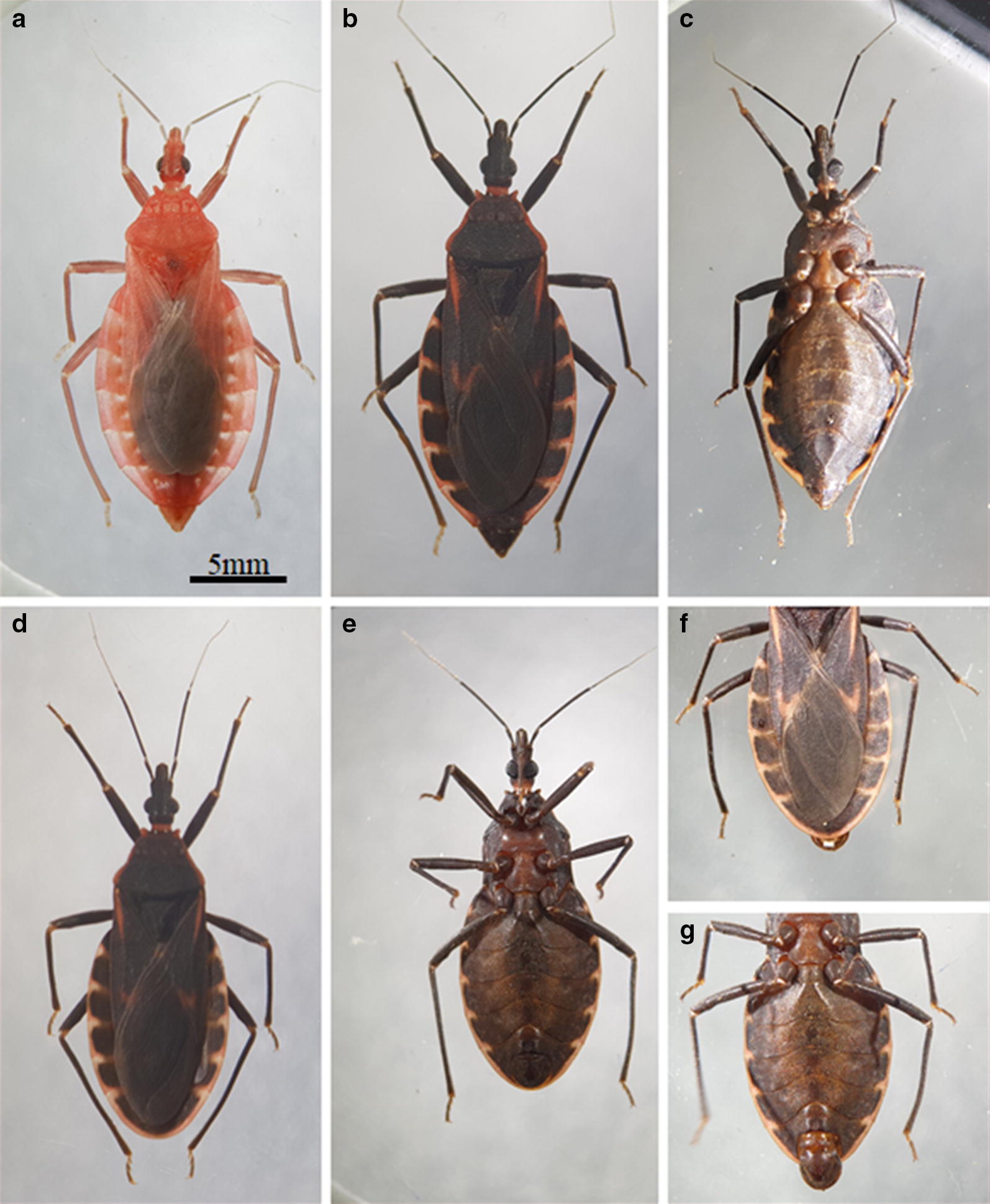

## Background

*Triatoma rubrofasciata* is a hemipteran insect of the subfamily Triatominae. There are around 150 triatomine species worldwide, most of them vectors of *Trypanosoma cruzi*, causative agent of Chagas disease in the Americas [1]. Of these species, only *T. rubrofasciata* is found in both New and Old Worlds [2], having been reported in many localities in Asia, Africa, the Americas (South, Central and North America), islands of the Indian Ocean, Pacific Ocean and the Azores in the Atlantic Ocean [1, 3]. However, in this century the occurrence of this species in the Americas has been restricted to coastal cities from Brazil. A national survey among 2004–2008 showed that in the Brazilian State of Maranhão, *T. rubrofasciata* represented more than 90% of the insects captured inside the homes [4]. In other South American countries such as French Guiana or Venezuela, *T. rubrofasciata* has not been found in the last 20 years [5]. On the contrary, the presence of *T. rubrofasciata* in Asia has increased in recent years, having been reported in China [6–8], Vietnam [3], India [9] and Sri Lanka [10]. In Vietnam, a national survey during the period 2010–2012 showed that *T. rubrofasciata* is found in at least 21 provinces, involving large cities such as Hanoi and Ho Chi Minh [3]. Urban infestations involve hundreds of insects per house, with a seasonal peak of adult bugs from June to September. Although *T. rubrofasciata* from Asia is not infected with *Tryp. cruzi*, it is frequently feeding on humans, causing severe bite reactions that sometimes lead to anaphylactic shock [3, 7–12].

In recent years, reports of bites of *T. rubrofasciata* to the human population have increased significantly in different places of Vietnam, becoming a public health problem [3, 13]. Bitten people present swelling and itching at the site of the bite, sometimes with a local skin infection. In Hanoi, 24% of the cases also involved a severe fever that lasted between one and two days [13]. Therefore, it is highly important to control *T. rubrofasciata* populations in order to avoid human attacks. For this reason, understanding of the biology of this species in Asia is indispensable [14].

*Trypanosoma conorhini* and *Tryp. lewisi* are extracellular kinetoplastid parasites of mammals, evolutionary close to *Tryp. cruzi* [15, 16]. Both are obligatory parasites of rodents of the genus *Rattus* with a worldwide distribution [17]. The cycle of *Tryp. conorhini* on *T. rubrofasciata* as vector has been documented long ago [17, 18]. Although there are no reports of human infection with *Tryp. conorhini*, several authors have demonstrated experimental infection in mice and non-human primates [19]. *Trypanosoma lewisi* is transmitted by rat fleas of genera *Nosopsyllus* and *Xenopsylla* [20] and traditionally it was considered as non-pathogenic to its natural hosts and humans [17]. However, several cases of human infection, including fatal ones, have been reported mainly in India [21–23] where *T. rubrofasciata* has been found [9]. As far as we know, this would be the first report of triatomine infection with *Tryp. lewisi.*

In the present study, *T. rubrofasciata* specimens were collected in several rural and urban areas from central Vietnam during 2014–2016. The collected individuals were identified as *T. rubrofasciata* following the morphological keys traditionally applied for triatomine species identification [1] as well as two genetic markers. We determined different biological attributes of Asian *T. rubrofasciata* and compared them with individuals from Brazil. Furthermore, a sample of one hundred individuals was scanned for natural infection with trypanosomatid parasites.

## Methods

### Triatomine collection and breeding

Triatomine bugs (891 eggs, 394 nymphs in all stages and 559 adults) were caught in both urban and rural areas from seven provinces in central Vietnam. The morphological and chromatic characteristics in the individuals collected were the same as those described in specimens from southern China [6–8]. Most of them were collected in two cities, Da Nang and Quy Nhon, during July 2014 to July 2016. The majority of the nymphs and adults were sacrificed for detection of parasites, while hatched eggs were used to establish the Da Nang laboratory colony. The laboratory conditions (humidity, temperature and light) were not controlled during the study. Bugs were kept in masked buckets and fed on live chickens weekly.

### Genetic studies

Cytogenetic analyses were performed on fourteen male *T. rubrofasciata* specimens collected from Da Nang city. Testes were removed from freshly killed adults, fixed in an ethanol:acetic acid mixture (3:1) and stored at − 20 °C. Chromosomal slides were obtained using the squashing method [24]. Briefly, small pieces of samples were transferred into 50% acetic acid drops. Squashes were made, the coverslips were removed, and the slides were left dried to proceed or stored at 4 °C. The C-banding technique [24] was used in order to determine the chromosome number and the distribution and behavior of C-heterochromatin during mitosis and meiosis. Fluorescent *in situ* hybridization (FISH) was carried out on 2 males [25] in order to determine the chromosomal location of *45S* ribosomal DNA clusters. Slides were analyzed using a Nikon Eclipse 80i epifluorescence microscope (Nikon, Tokyo, Japan). Images were captured with a Nikon DS-5Mc-U2 digital cooled camera using Nikon Nis Elements 3.1 Advanced Research software, and processed with Adobe Photoshop® software. The C-banding pattern for each specimen was determined by analyzing at least 10 cells, both in mitotic (spermatogonial prometaphase) and or meiotic plates (metaphase I or II).

Two adult individuals from Da Nang city were analyzed using the mitochondrial *cytochrome b* gene fragment (*cytb*). DNA was extracted from each individual using three legs fixed in 70% ethanol. Total DNA was isolated by standard phenol-chloroform techniques, resuspended in 50 µl and stored at − 20 °C until use. For each specimen, a *cytb* 561bp-fragment was PCR amplified [26]. PCR products were sent to Macrogen Inc. (Macrogen Inc., Seoul, Korea) for sequencing. For both samples, sequencing was conducted in forward and reverse directions. In addition, an NCBI BLAST search was performed in order to compare our samples with *T. rubrofasciata* sequences available on GenBank. Alignments were performed using MAFFT v7.310 [27]. To infer the basic population statistics and the genetic distances of the haplotypes, under Kimura 2-parameter model, we used R packages *Ape* [28] and *Pegas* [29]. First, all DNA sequences were loaded into R using read.dna function. Then, haplotypes were assessed using the haplotype function (nuc.div, hap.div and dist.dna for nucleotide diversity, haplotype diversity and genetic distances, respectively). Additionally, haplotype sequences were used to construct a network, using the function haploNet in the R package *Pegas* [29]. We plotted the haplotype network using the normal R plot function.

### Feeding capacity

To determine the amount of blood that an insect absorbs in each stage of its development, thirty insects from each nymphal instar were kept separated in cubes. Those insects that hatched or molted within three days were transferred to other cubes and weighed before and after their first meal after hatching or molting. The weight of the insects before and after feeding was measured and compared to assess feeding capacity (weights of taken bloods to hungry weights).

### Reproductive capacity

Ten adult couples were kept in separate cubes throughout their lives. The number of eggs produced by each couple every 24 h was recorded to calculate the minimum and maximum number of eggs produced by a single female in a single day and throughout her life. Ten couples of adults were randomly selected each month and kept as groups to investigate the seasonal-dependent reproductive capacity.

### Starvation resistance

After egg hatching (nymph first-instar) or molting (other stages), bugs were not provided food and were left to die. The date of hatching/molting and date of death were recorded to calculate starvation resistance.

### Size of bugs and life-cycles

The eggs that were produced and bugs that hatched or molted on same day were kept in separate buckets and fed weekly on live chickens until next molting or death. When the first bug in one group was undergoing molting to become a new nymph or adult, the size (width and length) of the other bugs in the same group was measured. In adult groups, when 50% of the females in one group produced eggs, the size of all members of the group was measured. Minimum and maximum time for egg maturation, development of each nymphal instar and living duration of adults were also recorded. The data of 30 nymphs at each instar and 60 adults (30 males and 30 females) were collected.

### Bug infection with *Trypanosoma* species

Microscope identification of *Tryp. conorhini* and *Tryp. lewisi* was made according to [18, 21], respectively.

## Results

### Collection sites of *T. rubrofasciata* in central Vietnam

All bugs (1844 including eggs) were caught in both urban (15 sites) and rural areas (7 sites). In 14 collection sites (9 urban and 5 rural) abundant bug nests were found including numerous eggs and bugs at all stages. In the remaining 8 sites (6 urban and 2 rural) only adults were found. In domestic environments, the beds, walls and roof cracks were the collection sites. In these habitats, no other food sources for insects were detected than the residents of the houses themselves. In urban and rural areas, peridomestic bug nests were found mostly in firewood storage places, wooden floors or cleft walls. In peridomestic structures, the bugs were mainly associated with rats (*Rattus norvegicus*) and in a smaller proportion with domestic mice. In rural environments, insects were also found associated with domestic animals such as cows or buffalos, pigs and chickens. In the regions of collection, triatomine bugs bites with anaphylactic shock have been reported. In all field trips to wild areas (far from human housing), we failed to collect bugs.

**Fig. 1:**
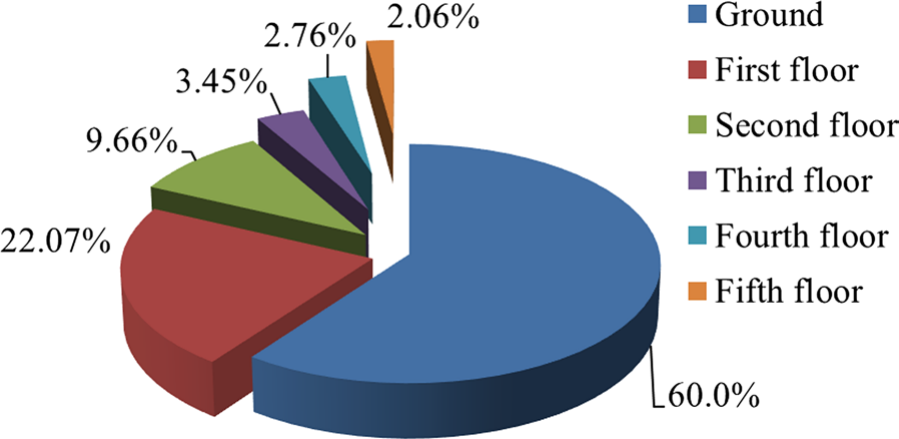
Scheme showing the distribution of individuals of *T. rubrofasciata* in urban dwellings collected on different floors

Of the 145 bugs collected inside houses (with people living there) in Da Nang city, 60% were captured on the ground floor, while only 22.07% of the bugs were captured on the first floor (Fig. 1). Few bugs were collected on second, third, fourth or fifth floors and none of them found on the sixth floor of the buildings. Additionally, abandoned houses and buildings provided a good environment for *T. rubrofasciata* within the city, with several very large nests including numerous eggs and bugs at all stages. The individuals collected from abandoned houses are not included in this study.

### Morphological and genetic identification

The individuals collected were identified as *T. rubrofasciata* following the morphological keys traditionally applied for triatomine species identification [1] with external characteristics similar to individuals from China [6–8].

**Fig. 2:**
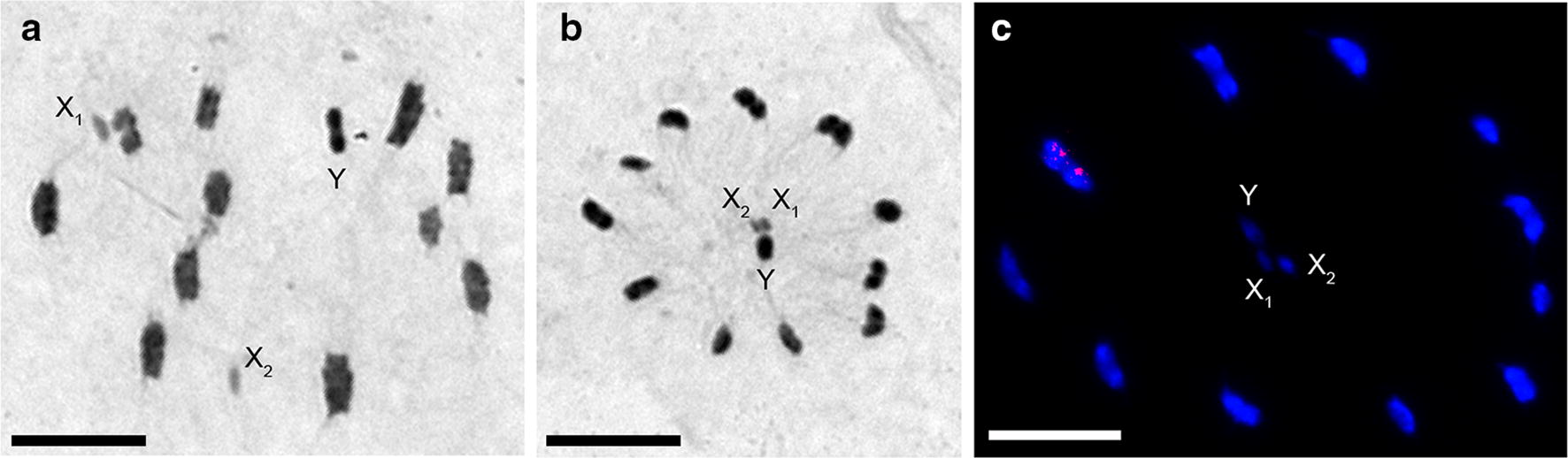
Meiotic chromosomes of *T. rubrofasciata* from Central Vietnam. **a** Metaphase I with C-banding. Eleven autosomal pairs and three sex chromosomes are clearly observed. **b** Metaphase II with C-banding. Typical spatial configuration of triatomines, with the autosomes forming a ring around the sex chromosomes that are arranged in the center. **c** Metaphase II. Fluorescent *in situ* hybridization with *45S* ribosomal DNA probe. The *45S* rDNA signals are located in the largest autosomal pair (red signals). *Scale-bars*: **a** 10 µm; **b** 10 µm; **c** 10 µm

Chromosomal analyses in all individuals showed a male diploid chromosome number of 25 chromosomes (22 autosomes plus 3 sex chromosomes: X_1_, X_2_ and Y) (Fig. 2). The eleven autosomal pairs had C-heterochromatic blocks in both chromosomal ends. The Y sex chromosome was totally heterochromatic, while the X_1_ and X_2_ sex chromosomes were the smallest of the complement (Fig. 2a, b). FISH technique indicated that *45S* ribosomal DNA clusters were located in the largest autosomal pair (Fig. 2c).

**Fig. 3:**
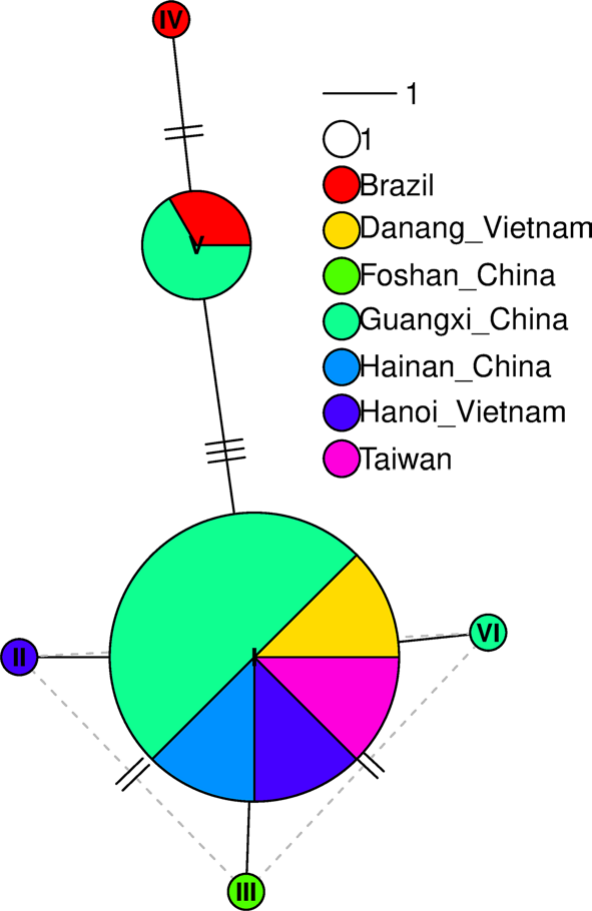
Haplotype network with 6 haplotypes identified from 16 sequences of *cytb* fragments. Pie charts are in proportional size with the frequency of the haplotype sequences, and branches are scaled according to the number of mutational steps, depicted with lines over the branches. The most frequent haplotype (I) is found in individuals from China (Guangxi, Hainan), Taiwan and Vietnam (Hanoi and Da Nang). Haplotype V is shared between individuals from Brazil and China (Guangxi)

The *cytb* sequences analyzed for both individuals from central Vietnam (Da Nang) were identical (GenBank: MN215889). Moreover, the BLAST search using these *cytb* sequences retrieved 14 matches, belonging to *T. rubrofasciata* individuals collected in China (several locations), Taiwan, Brazil and Vietnam (Hanoi). Considering the 14 sequences available on GenBank, plus the 2 individuals analyzed in this paper, a total of six haplotypes were identified (Fig. 3, Additional file 1: Tables S1, S2). In the 488 bp alignment, 8 sites were variable. The most frequent haplotype (I) was present in China (Guangxi, Hainan), Taiwan and Vietnam (Hanoi and Da Nang). Guangxi (China) was the location which presented most haplotype variation.

Nucleotide diversities were low, as expected for an intraspecific variation, and also comparable with the values usually reported for other triatomines (Additional file 1: Tables S1, S2). The largest genetic distance between *T. rubrofasciata* haplotypes was 0.013 between haplotype IV with haplotypes II, III and VI (Additional file 1: Table S2), as shown in Fig. 3. However, as this species possesses a trans-continental distribution, it is noteworthy that individuals located in Brazil and China (Guangxi) shared the same haplotype (V).

### Size, color and egg hatching

**Fig. 4:**
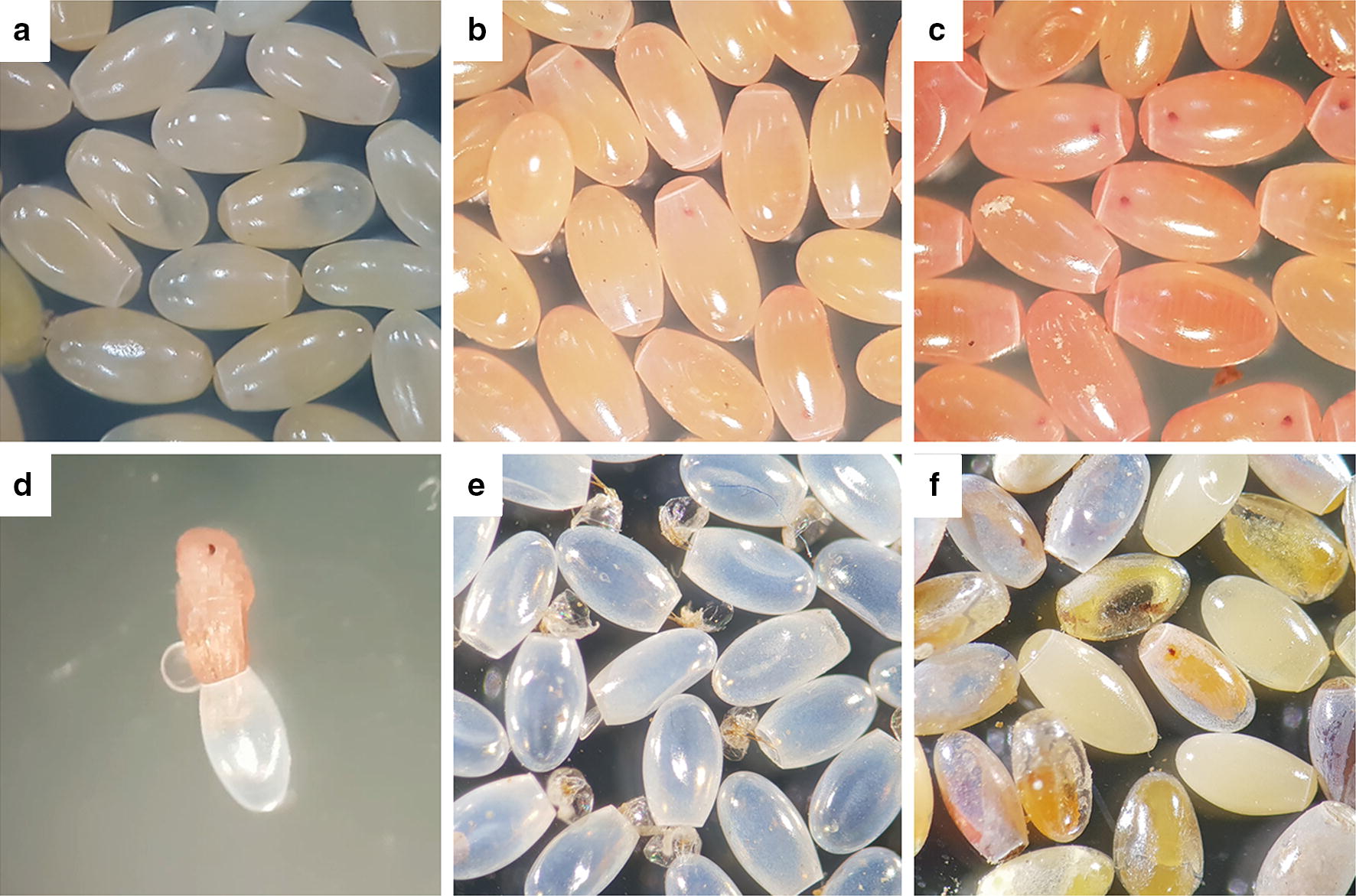
Eggs of *Triatoma rubrofasciata* before and after hatching. **a** Newly produced eggs. **b** Eggs in the middle of their development. **c** Eggs before hatching. **d** Nymph emerging from the egg. **e** Empty fertile eggs. **f** Non-viable eggs (without hatching)

The newly produced eggs were 1.79 ± 0.139 mm long and 0.91 ± 0.089 mm wide, and had a milky-white color (Fig. 4a). During their development, the eggs changed color to orange-yellow on days 3–4 (Fig. 4b) and to pink on day 7 (Fig. 4c). The eyes of the insects could be seen on day 3, and they turned reddish before hatching (day 10 onwards). At the time of hatching, the insect pushes the operculum open, enabling it to leave (Fig. 4d), leaving the egg shells transparent (Fig. 4e). A very low frequency of non-hatched eggs was detected due to unformed embryos or death of the embryo during its development (Fig. 4f). The eggs of *T. rubrofasciata* had a very high hatching rate; close to 98% (900 eggs, 882 nymphs of the first-instar). A female of *T. rubrofasciata* can produce eggs at any time of the year but the number of eggs gradually increased from January to July (on average 144 eggs per female), while it gradually decreased until reaching its lowest in December (on average 53 eggs per female). A single female can produce a minimum of 47 eggs and a maximum of 157 eggs.

### Life-cycles

**Fig. 5:**
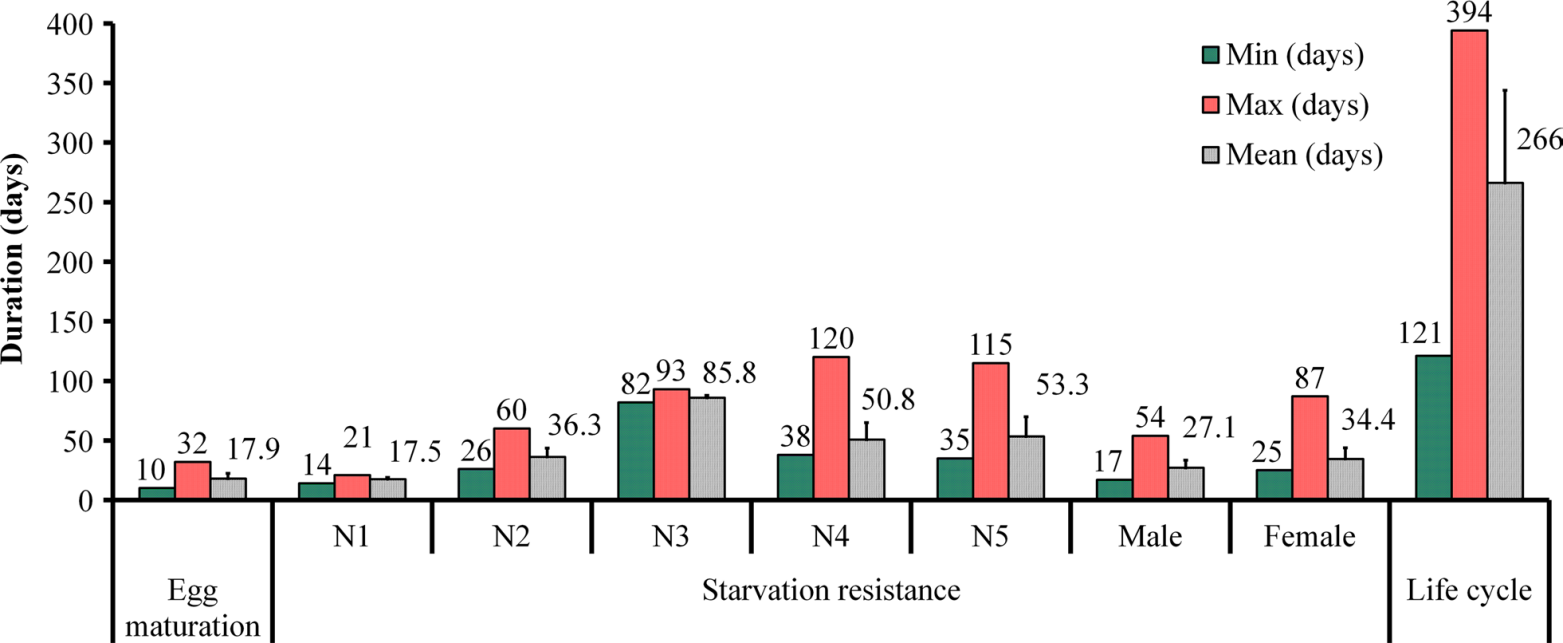
Starvation resistance of *T. rubrofasciata* from central Vietnam during their life-cycle

Figure 5 shows the minimum and maximum durations expressed in days for egg hatching and resistance to starvation of *T. rubrofasciata*. At least 10 days are required for eggs to hatch and a maximum of 32 days (lower temperatures during winter). Resistance to starvation increased in accordance with the stage of development, except for the adult bugs that presented resistance similar to the third-stage nymphs. After hatching, first-stage nymphs were able to survive for approximately 2–3 weeks without blood meals. Fourth-stage nymphs showed the most resistance to starvation, since they could stay for a minimum of 38 days and a maximum of 120 days after molting without food. The lowest resistance was observed in the first-stage nymphs and the longest in the third-stage nymphs (Fig. 5). Life-cycles (newly produced eggs until death of adults) varied from 121 to 394 days. In addition, a minimum of 82 days and a maximum of 256 days were required for viable eggs to reach adulthood.

### Mortality rate of each nymphal stage

**Fig. 6:**
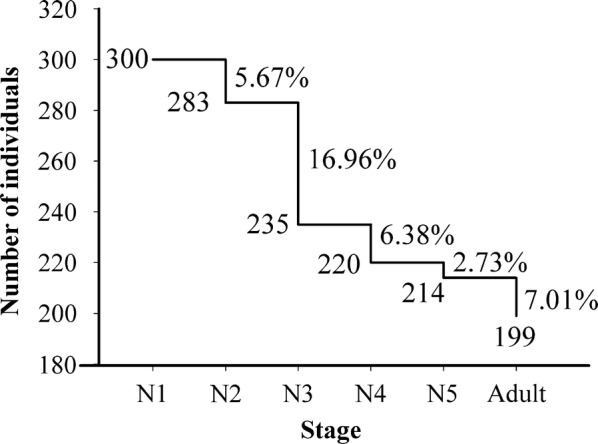
Mortality rate in all nymph stages of *T. rubrofasciata* from central Vietnam. Numbers indicate initial number of bugs, and percentages indicate mortality rate before molting

The mortality rate of each nymphal stage was analyzed from an initial population of 300 first-stage nymphs. The mortality rate of each nymphal stage was relatively low, always lower than 10% except for the second- to the third-instar (17%) (Fig. 6). An accumulated mortality rate of 33.7% was observed from the first-instar to the adult.

### Feeding capacity

**Fig. 7:**
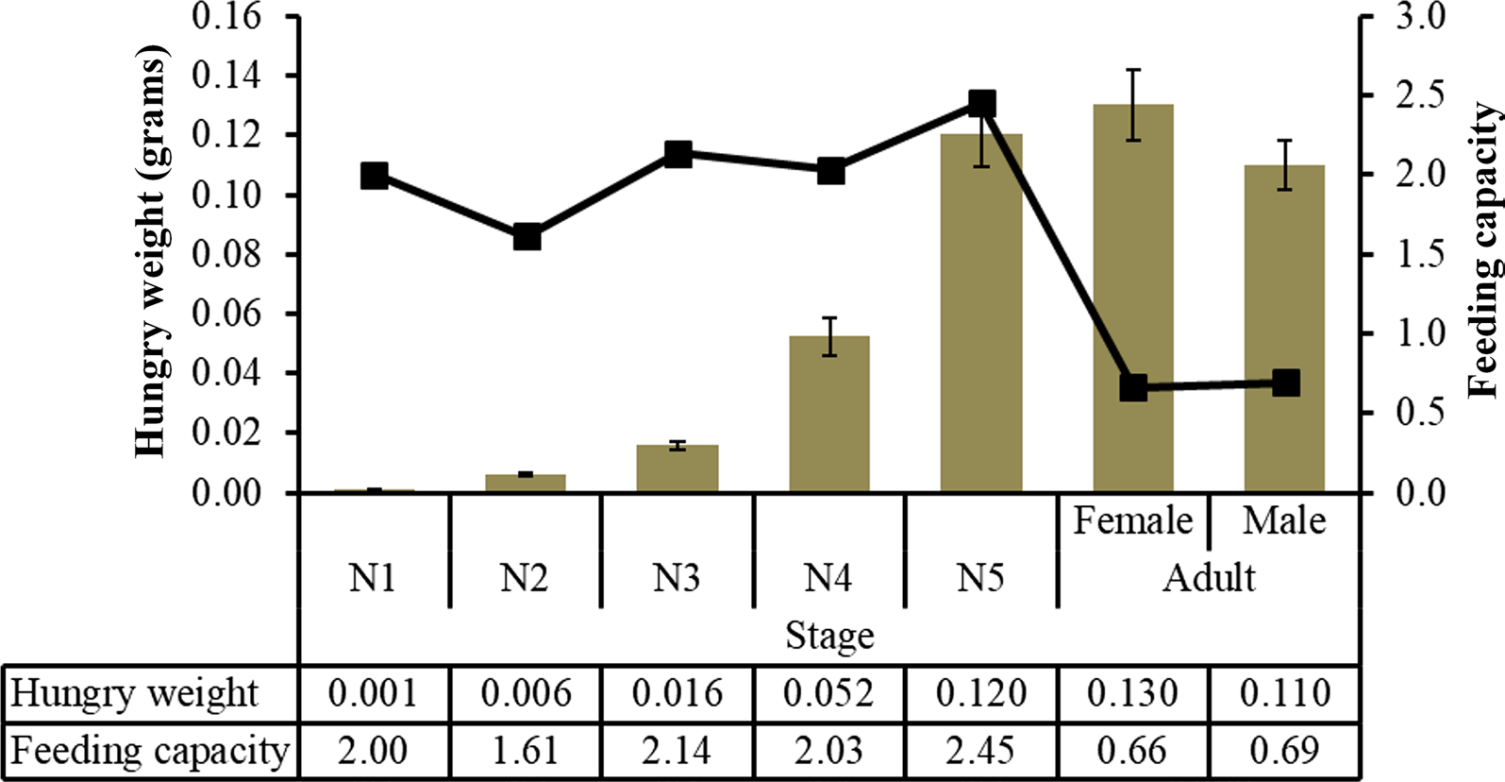
Bugsʼ unfed weight (bars) and ratios of blood weights in first meal of bugs compared to their own unfed weight (line)

The weight of blood that nymphs could ingest in a single meal was greater than their own unfed weight (Fig. 7). All nymph stages increased almost thrice or more their own weight, specifically nymphs of the fifth-instar which presented a greater feeding capacity. In adults, regardless of sex, this relationship decreases significantly, representing only 68% of their own weight.

### Natural and experimental infection with *Trypanosoma* species

A total of 100 adult bugs collected in domestic and peridomestic environments were examined. Of these, 37 were infected with *Tryp. conorhini* only, 18 were infected with *Tryp. lewisi* only and 9 were infected with both parasites. In conclusion, 64% of the insects examined were infected by at least one species of *Trypanosoma*. On the other hand, 180 nymphs in the first-stage (N1), divided into 6 groups, were experimentally infected with *Tryp. conorhini* and *Tryp. lewisi* by feeding them on infected mice in a single meal. Then the insects continued to be fed on live chickens once a week until they were sacrificed. The first group (N1) was sacrificed one week after the initial infection and each of the 5 remaining groups were sacrificed when they reached the different nymphal stages (N2, N3, N4 and N5) and adulthood, respectively. The parasite analysis of each group showed that 100% of the insects kept the parasites throughout their lives.

## Discussion

*Triatoma rubrofasciata* presents several characteristics in the antennas, head and thorax that make them easily distinguishable from the other triatomine species [1]. In addition to corroborating the presence of these traits, we used genetic markers to confirm that the individuals collected in central Vietnam belong to *T. rubrofasciata*. At the chromosomal level, of almost 100 triatomine species cytogenetically described [30], only *T. rubrofasciata* has 22 autosomes, which we observed in all of the individuals analyzed (Fig. 2). Other chromosomal characteristics, such as the position, size and amount of C-heterochromatin, and the chromosomal position of ribosomal clusters are similar in individuals from Brazil and Vietnam (south and central) (present study and [13]).

By analyzing a partial fragment of the mitochondrial *cytb* gene, including all sequences available on GenBank [6, 13, 31] and the two individuals here studied, we were able to determine the existence of six haplotypes corresponding to 16 individuals (4 from Vietnam, 2 from Brazil, 1 from Taiwan, 9 from South China) (Fig. 3). These haplotypes exhibit very low genetic distances (Additional file 1: Tables S1, S2), being compatible with expected levels of intraspecific variation. Furthermore, one of these haplotypes was shared between China and Brazil. This can be only explained by recent migration events. Tropicopolitan distribution of *T. rubrofasciata* is mainly explained by migration associated with their host, *Rattus rattus*, on commercial ships during the last 500 years [32–35]. Two very different hypotheses have been formulated about the evolutionary origin of *T. rubrofasciata* [3, 32–36]. The first hypothesis suggests that all Old World triatomines descend from a common ancestor that arrived in Eurasia from North America through the Bering Land Bridge about 20 million years ago. Therefore, *T. rubrofasciata* would have an Asian origin and its presence in the Americas would be the result of recent migrations [3, 36]. The alternative hypothesis is that all Old World triatomine species derive from a single modern species, *T. rubrofasciata*, which was transported from the Americas to Asia in commercial ships from the 16th–17th centuries [3, 32–35]. Our data with *cytb* sequences showed a high genetic similarity suggesting a common and recent origin of the Asian (Vietnam, China) and American populations (Brazil). Similar results were recently shown using other nuclear (*28S* rDNA) and mitochondrial (*16S* rDNA and *cox*1*)* markers [8]. Furthermore, the low genetic variation between *Tryp. conorhini* from Brazil and Hawaii also supports a recent migration event [37]. An extensive population analysis which includes individuals from other regions such as India, Indonesia as well as the Caribbean islands would be necessary in order to evaluate the two hypotheses (Asian *versus* American) regarding the origin of *T. rubrofasciata* [36].

In central Vietnam, *T. rubrofasciata* is present in domestic environments and their surroundings, and apparently absent in wild habitats despite the various searches that were carried out. Until now, *T. rubrofasciata* has never been reported occupying wild habitats in both the Americas and Asia [36]. In big cities, this species has the capacity to inhabit houses of good construction quality, having the inhabitants as the main source of food. In these cases, the insects are concentrated in the lower floors of the buildings (82% on the ground and first floors), probably due to their limited flight capacity (Fig. 1). In peridomestic habitats, both in urban and rural areas, *T. rubrofasciata* has always been associated with wood, both in firewood storage places as well as walls or ceilings of human constructions. All stages of the bugs were collected throughout the year revealing that their reproductive activity is not restricted to a particular season.

The average and maximum number of eggs that a female of *T. rubrofasciata* from central Vietnam produces (47 and 157 eggs, respectively) were lower than those reported for the species in Brazil (243 and 410 eggs, respectively) under similar feeding conditions [38]. However, the minimum time for eggs to hatch was 10 days, shorter than that reported for *T. rubrofasciata* from Brazil (16 days) and for other triatomine species [39]. These differences could be due to uncontrolled environmental conditions in our laboratory, especially in summer with temperatures above 35 °C and relative humidity greater than 75%. From the study of 900 eggs, the hatching rate was almost 98%, a value much higher than that observed in individuals from Brazil (80.5%) [38] or in other evolutionarily close species such as *T. gerstaeckeri* (75%), *T. lecticularia* (82.5%) and *T. protracta* (76%) [39].

From the analysis of 300 nymphs in the first stage (N1), 199 adults were obtained, with a mortality rate of 33.7% (Fig. 6). This mortality is a little higher than that observed in *T. rubrofasciata* from Brazil (24.6%) where 179 N1 resulted in 135 adults [38]. The time required from hatching of eggs to adult varies between 82 and 256 days, very similar to those observed in Brazil (83 and 246 days). Comparatively with other triatomines species, *T. rubrofasciata* (found in Vietnam and Brazil) requires much less time to complete its life-cycle than other species of triatomines, such as *Meccus picturatus* (173 days), which feeds on chickens, or *T. gerstaeckeri* (255 days), *T. lecticularia* (201 days) and *T. protracta* (196 days) which feed on rabbits [39, 40].

Our results on resistance to starvation (Fig. 5) show that the specimens from Vietnam have a greater fasting capacity than Brazilian specimens [41]. However, this comparison should be considered with caution since the breeding conditions, especially the food sources, were different (chickens in Vietnam and rats in Brazil). The nymphal stages have maximum periods of starvation between 60 and 120 days (except for nymph stage 1 with 3 weeks). These long periods would facilitate their dispersion by passive transport and the persistence of infestations in domiciles as well as in temporarily abandoned dwellings, similar to that described in *R. prolixus* [42].

The blood-feeding capabilities of the nymphs were surprisingly high (Fig. 7). Similar to the data reported for *T. rubrofasciata* in India [43], each nymphal instar increased its own weight two-fold, with the fifth-instar showing the greatest increase in weight (Fig. 7), which is typical in insects [44]. In adults, regardless of sex, this relationship decreased significantly, representing only 68% of their own weight. In addition, insects were able to survive for a long time without food. The values of blood meal in adults were similar to that reported for *T. patagonica* (65% of their weight for females) [45]. Similar to Brazilian [46] and Indian *T. rubrofasciata* [47], this species from Vietnam showed natural infections with *Tryp. conorhini* (46%). To the best of our knowledge, this study reports for the first time a natural infection of triatomine insects with *Tryp. lewisi* (27%), a pathogenic parasite for humans which could represent an important burden on human health [21]. *Triatoma rubrofasciata* had a very high natural infection rate with 64% of the bugs infected by at least one *Trypanosoma* species.

## Conclusions

This study showed that *T. rubrofasciata* has the capacity to invade human dwellings in urban areas of big cities in central Vietnam, using the inhabitants of the houses as a food source. In peridomestic environments of urban and rural areas, this species is mainly associated with wood structures and stored wood, which are very common in several Asian countries. *Triatoma rubrofasciata* has a fairly short life-cycle under uncontrolled laboratory environmental conditions, probably very similar to those found in their natural habitats. Their high rate of fecundity throughout the year, high capacity for starvation, as well as the occurrence of this species in synanthropic environments with high availability of food sources (in the case of Vietnam, rats, mice and chickens) are risk factors to be considered by vector control campaigns. Finally, several allergic reactions caused by the bites of *T. rubrofasciata* and their high infection with *Tryp. lewisi* underline the importance of analyzing its vector role and impact on human health.

## Supplementary information

**Additional file 1: Table S1.** Basic population statistics of *T. rubrofasciata* sequences available on GenBank. *Abbreviations*: Nh, number of haplotypes; Hd, haplotype diversity; Nucl. div., nucleotide diversity (π). **Table S2.** Genetic distances among the 6 haplotypes of *T. rubrofasciata* worldwide, calculated under Kimura 2-parameter model.

## Data Availability

All relevant data are within the article and its additional file.

## Abbreviations

*cox*1: cytochrome *c* oxidase subunit 1 gene
*cytb*: *cytochrome b* gene
PCR: polymerase chain reaction
