# Supplementary material for: Biological attributes of the kissing bug Triatoma rubrofasciata from Vietnam
Source: Parasit Vectors. 2019 Dec 16;12:585. doi: 10.1186/s13071-019-3844-6 (PMC6915989; doi:10.1186/s13071-019-3844-6)
Supplement: Supplementary file 1 — Additional file 1: Table S1. Basic population statistics of T. rubrofasciata sequences available on GenBank. Abbreviations: Nh, number of haplotypes; Hd, haplotype diversity; Nucl. div., nucleotide diversity (π). Table S2. Genetic distances among the 6 haplotypes of T. rubrofasciata worldwide, calculated under Kimura 2-parameter model. [file 13071_2019_3844_MOESM1_ESM.doc]

**Additional file 1: Table S1. Basic population statistics of *T. rubrofasciata* sequences available on GenBank**

| Countries | Sequences | Nh | Hd | Nucl. div. (π) |
| --- | --- | --- | --- | --- |
| Brazil | 2 | 2 | 1 | 0.004098361 |
| China | 9 | 4 | 0.6944444 | 0.003301457 |
| Vietnam | 4 | 2 | 0.6666667 | 0.00136612 |
| Taiwan | 1 | 1 | --- | --- |

Nh: Number of haplotypes. Hd: haplotype diversity. Nucl. div.: Nucleotide diversity (π).

**Additional file 1: Table S2. Genetic distances among the 6 haplotypes of *T. rubrofasciata* worldwide, calculated under Kimura 2 parameters method**

|  | I | II | III | IV | V |
| --- | --- | --- | --- | --- | --- |
| Haplotype II | 0.002 | 0 |  |  |  |
| Haplotype III | 0.002 | 0.004 | 0 |  |  |
| Haplotype IV | 0.010 | 0.013 | 0.013 | 0 |  |
| Haplotype V | 0.006 | 0.008 | 0.008 | 0.004 | 0 |
| Haplotype VI | 0.002 | 0.004 | 0.004 | 0.013 | 0.008 |
